# Validity and Reliability of Persian Smell Identification Test 

**DOI:** 10.22038/ijorl.2019.35782.2180

**Published:** 2020-03

**Authors:** Seyed Kamran Kamrava, Maryam Jalessi, Sahand Ghalehbaghi, Elahe Amini, Rafieh Alizadeh, Farhad Rafiei, Sadaf Moosa, Mohammad Farhadi

**Affiliations:** 1 *ENT and Head and Neck Research Center and Department ,Hazrat Rasoul Hospital, the Five Senses Institute, Iran University of Medical Sciences, Tehran, Iran.*; 2 *Skull Base Research Center, Hazrat Rasoul Haspital, the Five Senses Institute, Iran University of Medical Sciences, Tehran, Iran.*

**Keywords:** Identification, Iran, Olfaction Disorder, Smell

## Abstract

**Introduction:**

Smell Identification Tests (SIT) are routinely utilized for the clinical evaluation of olfactory function. Since Iran consists of various ethnic subgroups, the reliability and validity of this test as a national SIT are required to be evaluated across the country.

**Materials and Methods:**

This cross-sectional study evaluated the cultural adaptation of SIT administered to 420 healthy volunteers from 6 various ethnic subgroups (i.e., Fars, Turk, Kurd, Lor, Baluch, and Arab) living in 7 cities (one city for each subgroup, and Tehran [capital of Iran] with mixed ethnicities). The SIT consists of pens pre-filled with 24 odorants. The correct identification response rate was evaluated in all and each subgroup. The test was performed twice on 60 participants with a 2-week interval to assess its reliability. The SIT was further administered to 150 cases with documented abnormal olfactory function to evaluate its validity.

**Results:**

The correct identification response rate was estimated at 70% for all odorants in all and each subgroup. The mean odor identification score was 21.41±1.37 (score range: 17- 24) with no significant difference among various subgroups. Moreover, the test-retest correlation coefficient was obtained at 0.77. The mean odor identification score in patients with olfactory impairment was 10.69±3.76, which was significantly different from that in healthy participants (P<0.001). The best cut-point for the beginning of olfactory impairment was 17.5 (95% CI: 9-100, Sensitivity=99, Specificity=81). Females obtained higher scores of odor identification, compared to males (P=0.025).

**Conclusion:**

The results indicated the reliability and validity of the SIT, which can be used nationally for the assessment of olfactory function in various ethnic subgroups across the country.

## Introduction

Although olfactory impairment may not be noticed as an apparent disability, it can severely affect the quality of life (1,2). Recently, developed tests for the clinical assessment of olfactory function have revealed some hidden associations between olfactory impairment and other diseases, the prominent example of which is the early diagnosis of neurodegenerative diseases, including Alzheimer's and Parkinson's diseases(3,4).

Among various kinds of tools used for the assessment of olfactory function, psychophysical instruments, including Smell Identification Test (SIT), was more popular due to its availability and simplicity of use in clinics (5). The odorants are the key elements for the assessment of olfactory dysfunction in SIT. However, the familiarity of the respondents with the odorants is an issue that interferes with adopting a uniform olfactory test (6,7). Therefore, it is necessary to assess and adopt a national valid and reliable SIT in each country (6-8). Popular olfactory tests, such as the University of Pennsylvania Smell Identification Test (UPSIT) and Sniffin’ Stick, have been validated for use in some other countries. Although the UPSIT is one of the best-validated tests in some countries, it is not suitable for the assessment of olfactory impairments in Iran since the Iranian population is unfamiliar with 24 odorants out of 40 fragrances (9).

 In a preliminary study, 24 culturally-familiar odorants were presented to a group of Iranian people (10). However, since Iran consists of various ethnic subgroups, it is necessary that the Persian SIT (PSIT) be evaluated in various parts of the country. This study aimed to evaluate the cultural adaptation along with the validity and reliability of this test across the country. 

## Materials and Methods

This cross-sectional study was performed from September 2015 to August 2016 in three phases. Initially, 6 cities including the most popular ethnic subgroups (i.e., Fars, Turk, Kurd, Lor, Baluch, and Arab) were randomly selected from the list of cities dedicated to each subgroup (based on the data from Iran’s national portal of electronic services, the Ministry of Information and Communications Technology). People who referred to the health clinics (age range: 20-60 years) were asked whether they had a normal sense of smell or not. In case they declared no olfactory impairments, they were requested to voluntarily participate in a free test of olfactory function. These participants were evaluated by general practitioners. The exclusion criteria were: 1) a history of chronic sinonasal, mental, psychological, or any chronic systemic diseases, 2) previous sinonasal surgery, 3) the consumption of medications that might affect the olfactory function, 4) head trauma, 5) recent upper respiratory tract infection, and 6) allergy. 

The PSIT used in this study included 24 culturally-familiar odorants (Magnolia Co., Iran), including Coffee, Vinegar, Banana, Mint, Coconut, Garlic, Curd, Apple, Cinnamon, Menthol, Cucumbers, Pineapple, Lemon, Orange, Saffron, Smoke, Rosewater, Cardamom, Honey, Vanilla, Hazelnut, Cantaloupe, Butter, and Onion. It has been revealed that the Iranian people are familiar with these fragrances (10). Minimum identification concentration (MIC) of each odorant has been evaluated and defined according to a previous study; in addition, the pre-filled pens and odorants were provided by Nanobon Company and a local fragrance supplier company named Magnolia (10), respectively. The odorants were presented in uniform pens with tampons filled with odorants that had been labeled with different codes at the bottom. 

The evaluations were performed at well-ventilated quite rooms in each city by physicians who were informed of the principles of the test and research procedure.

The physicians were required to remove the cap of each pen and placed it in 1 to 2 cm in front of the nostrils of the participants. Subsequently, the participants were asked to sniff the odorant and mark an option from a list of 3 choices. The whole process was repeated for all 24 odorants with 30-sec intervals. For the assessment of cultural adaptation, the correct identification response rate (%) for each odorant and the odor identification scores were evaluated in all and each ethnic subgroup.

In the next phase, 10 participants from each city were randomly asked to repeat the test after a 14-days interval. The correlation between the results of these two sets of responses was evaluated to assess the reliability of the SIT. Totally, a 2-week interval was selected to minimize the effect of weather changes on the olfactory function.

Eventually, the patients who referred to the clinic with a complaint of nasal and sinus problems were asked about their sense of smell. Those with hyposmia or anosmia were requested to voluntarily participate in the study. They were not involved in any litigation according to the olfactory function. In total, 150 patients with olfactory dysfunction based on the Sniff Magnitude Test results were included in this study (the mean proportion of the area under the curve for each odor was higher than 0.80) (11,12). These patients were further evaluated by the SIT. The mean odor identification score obtained from this group was compared to that of the healthy participants to evaluate the validity of the SIT. The study protocol was approved by the Ethics Committee of the Ear, Nose, and Throat and Head and Neck Research Center, Iran, and the written informed consent was obtained from the participants. It should be noted that all research procedures were in accordance with the Declaration of Helsinki. The data were analyzed in SPSS software (version 22.0, Inc., Chicago, IL) through the Pearson correlation coefficient, one-way ANOVA, and the Mann-Whitney U test. Moreover, the quantitative and categorical variables were presented as mean±SD and frequency, respectively. A P-value less than 0.05 was considered statistically significant.

## Results

At the first stage, 420 healthy volunteers (60 subjects from each city with a mean age of 36.84±10.27 years [age range: 20-60 years]) were evaluated to assess the cultural adaptation of 24 previously defined odorants. It should be noted that half of the participants were male. The correct identification rate (%) for all and each ethnic subgroup was determined and illustrated in Table 1, which was determined at 70% in this study. According to the results, there were no significant differences among the ethnic subgroups regarding the correct identification rate.

The mean odor identification score was 21.41±1.37 (score range: 17-24) which was not significantly different among the ethnic subgroups. Females obtained significantly higher mean identification scores, compared to males (21.56±1.26 vs. 21.26±1.48, P=0.02). Table 2 summarizes the mean odor identification scores in all and each ethnic subgroup. Within each ethnic subgroup (i.e., Fars and Arab volunteers), a significant difference was observed between males and females in terms of the mean identification scores. However, there were no significant differences between males and females in Lor, Tork, Kord, Baloch, and mixed ethnic subgroups in this regard.

**Table 1:** Correct identification rate (%) obtained from 420 cases and each subgroup

**Odorants**	**Mixed**	**Kurd**	**Baluch**	**Lur**	**Turk**	**Arab**	**Fars**
Smoke	98.3%	98.3%	96.7%	98.3%	96.7%	96.7%	95%
Vanilla	81.7%	83.3%	83.3%	85%	86.7%	78.3%	88.3%
Butter	88.3%	81.7%	85%	86.7%	88.3%	86.7%	90%
Hazelnut	95%	96.7%	93.3%	86.7%	95%	86.7%	93.3%
Rose	85%	91.7%	85%	86.7%	88.3%	83.3%	90%
Cardamom	85%	90%	80%	86.7%	81.7%	81.7%	88.3%
Saffron	88.3%	86.7%	93.3%	86.7%	90%	91.7%	91.7%
Coffee	91.7%	96.7%	95%	98.3%	98.3%	93.3%	91.7%
Cinnamon	98.3%	96.7%	95%	95%	91.7%	93.3%	93.3%
Banana	93.3%	86.7%	91.7%	95%	98.3%	95%	96.8%
Garlic	91.7%	95%	96.7%	91.7%	91.7%	95%	93.3%
Onion	91.7%	95%	93.3%	96.7%	91.7%	90%	93.3%
Mint	90%	90%	95%	91.7%	95%	93.3%	95%
Cantaloupe	91.7%	91.7%	91.7%	91.7%	91.7%	91.7%	91.7%
Honey	91.7%	86.7%	90%	96.7%	90%	93.3%	86.7%
Apple	91.7%	91.7%	90%	93.3%	88.3%	88.3%	91.7%
Pineapple	76.7%	85%	78.3%	80%	83.3%	93. 3%	85%
Vinegar	85%	90%	93.3%	86.7%	86.7%	85%	86.7%
Coconut	90%	90%	93.3%	90%	88.3%	85%	85%
Curd	78.3%	81.7%	86.7%	86.7%	75%	86.7%	81.7%
Vicks	78.3%	73.3%	71.7%	75%	71.7%	78.3%	80%
Cucumber	91.7%	88.3%	90%	88.3%	88.3%	88.3%	86.7%
Orange	90%	90%	86.7%	88.3%	88.3%	91.7%	88.3%
Lemon	76.7%	85%	78.3%	80%	83.3%	86.7%	85%

**Table 2:** Mean ±SD of identification scores among various ethnic subgroups

**Ethnicity**	**Mean±SD** **Total**	**Mean±SD** **Males**	**Mean±SD** **Females**	**P**-**Value**
Fars	21.48±1.396	21.00±1.554	21.97±1.033	0.006
Turk	21.35±1.162	21.07±1.143	21.63±1.129	0.049
Kurd	21.47±1.535	21.30±1.784	21.63±1.245	0.405
Lor	21.45±1.156	21.47±1.383	21.43±0.898	0.912
Baluch	21.42±1.394	21.53±1.408	21.30±1.393	0.521
Arab	21.37±1.426	21.00±1.486	21.73±1.285	0.045
Mixed ethnicities	21.32±1.578	21.43±1.547	21.20±1.627	0.571
Total	21.41±1.37	21.26±1.477	21.56±1.256	0.025

 The area under the distribution curve was considered as a parameter to define the normal range. The mean and mode values of the identification scores were estimated at 21. Totally, 78.1% of the volunteers obtained an identification score of 21 and higher. Following that, the reliability of the test was evaluated according to the results of the SIT on 60 cases who had taken it twice. The test-retest correlation coefficient of the test was obtained at 0.77. At the final stage, the mean identification score was evaluated based on the test results of 150 patients with hyposmia/anosmia to assess the validity of the test. The identification score of the patient group was determined at 10.69±3.76, which was significantly lower than that obtained from the healthy participants (P<0.001). The best cut-point for the beginning of olfactory impairment was 17.5 (95% CI: 99-100, Sensitivity=99%, Specificity=81%), whereas the corresponding point was 18.5 in this study (Sensitivity=97%, Specificity=81%).

## Discussion

The SITs are among the commonly-used tools for the assessment of the olfactory function in patients with a normal or impaired sense of smell. One of the popular SITs belongs to the UPSIT that was introduced in the early 1980s by Doty et al. (13). After realizing the role of cultural familiarity in adopting the olfactory tests, several modified versions along with the international versions of UPSIT were developed in various countries (e.g. Japan, Turkey, Brazil) (7,14-16). 

Overall, the correct identification rate of more than 70% is needed for an odorant to be adopted in SITs(7,17,18). In a previous study performed by Kamrava et al. (9), it was shown that the Iranian population was not familiar with the UPSIT; therefore, it was necessary to develop a reliable and valid test. Similarly, another study evaluated 24 culturally-familiar odorants and their MICs in Iran (10). 

Kondo et al. evaluated the original version of UPSIT in Japan (19). According to the results of the aforementioned study, this test was not suitable to be administered to the Japanese population since they were not familiar with 10 items of the original version of UPSIT. Ogihara et al. (14) revised this test in a subsequent study, and when they introduced this new version across the country, 9 items obtained a correct identification rate of <80%. Therefore, various ethnic or socio-cultural groups, even inside a country, might not be equally familiar with the odorants. In our first study, the odorants in the UPSIT were evaluated to find out the familiar fragrances (9). Subsequently, in the second study, 24 odors were selected while assessing their MIC (10). Surprisingly, according to the results of our first study in which Iran-SIT was used, the odors, such as "Grape" and "Strawberry", obtained the familiarity rates of 13.3% and 55%, respectively (9).

Since Iran consists of various ethnic and cultural subgroups and Iran-SIT evaluated only a healthy population in Tehran (i.e., capital of Iran), not across the country, it is necessary to develop a national SIT to be utilized countrywide or at least in cities including the most popular ethnic subgroups.

In another study carried out by Taherkhani et al. (20), a version of UPSIT was administered to the healthy population in Tehran, Iran, not across the country. It is worth mentioning that the validity of this test was not assessed in the aforementioned study. In our first study, "Grape" and "Strawberry" odors that were used in the Scratch SIT obtained the familiarity rates of 13.3% and 55%, respectively (9). The utilization of scratch-and-sniff stickers requires related technology that may not be available in some parts of our country. The cost of scratch-and-sniff stickers may prevent their use for the public. While the smelly pens can be used frequently, scratch-and-sniff stickers can be utilized only once.

There are different ways to deliver an odorant in an SIT, and pre-filled pens were utilized in the present study. While the UPSIT uses "Scratch and Sniff", the Sniffin’ Sticks test utilizes pens pre-filled with various odorants (7,13-21). The original Sniffin’ Sticks test was presented with 16 odorants by Hummel et al. (21) in Germany during 1997 for assessing the threshold, discrimination, and identification. However, the cultural unfamiliarity was shown to be an issue for adopting original Sniffin’ Sticks as an SIT in other countries. Konstantinidis et al. (8) demonstrated that the Greek population was not familiar with 6 items of a standard version of Sniffin’ Sticks (correct identification rate of <70%).

Italy has also adopted a modified version of the Sniffin’ Sticks. Other countries, such as Australia, Sri Lanka, Brazil, and Taiwan have modified the test cross-culturally (22-24). In a recent study carried out by Oleszkiewicz et al. (25), 24 culturally-familiar odorants have been presented in the form of pre-filled pens to the Egyptian population. Sniffin’ Sticks tests are cost-benefit and reusable with a lifespan of approximately 6-12 months, compared to the UPSIT (16). However, this test cannot be sent via mail to the patient, which is a superiority of the UPSIT. As a result, for a more accurate evaluation, an expert may be needed to administer the test. 

There are studies considering the effect of age on olfactory function(8,26). The majority of these studies believed that patients younger than 20 and older than 60 years of age had lower olfactory abilities, compared to the ones aged 20­60 years (8,26). Therefore, these two age spectrums were not included in the present study. Furthermore, some authors indicated that sex hormones had the ability to affect the olfactory function. In a systematic review performed by Doty et al.(27), females were shown to have more correct identification rate regarding some odorants, especially body odorants, compared to males. The females also obtained higher UPSIT median scores, which were in line with the findings in this study. With a statistically significant difference, it was revealed that females were more sensitive to odorants, including coffee (P=0.004) and mint (P=0.049). However, the conditions, such as pregnancy and the consumption of hormonal contraceptives should be considered for more accurate analysis (28-30). 

In the present study, test-retest correlation coefficients were obtained at 0.77, which was higher than that obtained from a modified Portuguese version (0.62) and close to those in a study conducted in Taiwan (0.76) or original German ones (0.73)(21, 31). However, these scores were less than the test-retest correlation efficiency of 0.92 obtained from UPSIT (13). The reliability of an SIT can be affected by the number of odorants. The original Sniffin’ Sticks test achieved the correlation efficiency of 0.73 with 16 odorants, compared to our PSIT with 24 odorants. The reason may be attributed to the cultural variety of the population in this study. Although the best cut-point for the beginning of olfactory impairment was 17.5, in practice, the cut-point should be considered at 18 since the results from SIT are discrete numbers. That is why the sensitivity and specificity of the next number (18.5) are mentioned in this study. It means that if 18 is considered as the cut-point for the beginning of olfactory impairment, the sensitivity of the test will be more than 97%. Although the familiarity rate of the odorants used in this study had been evaluated previously, a randomized selection of various ethnic subgroups out of the Iranian population is required for the validation of the PSIT. 

## Conclusion 

The PSIT was revealed to be a reliable and valid test. It decreased the interference of various ethnics in olfactory assessment and can be used nationwide. All odorants used in this test obtained an acceptable familiarity rate with no obvious differences among the ethnic subgroups.
